# Analytical Validation and Clinical Implementation of a 1080-Gene Comprehensive Genomic Profiling Assay with Integrated Cloud-Based Analysis for Solid Tumor Molecular Oncology

**DOI:** 10.3390/biomedicines14071462

**Published:** 2026-06-27

**Authors:** Ashutosh Vashisht, Ashis K. Mondal, Vishakha Vashisht, Pankaj K. Ahluwalia, Saloni Andhari, Jaspreet Farmaha, Jana Woodall, Ravindra Kolhe

**Affiliations:** Department of Pathology, Medical College of Georgia at Augusta University, Augusta, GA 30912, USA; avashisht@augusta.edu (A.V.); amondal@augusta.edu (A.K.M.); vvashisht@augusta.edu (V.V.); pahluwalia@augusta.edu (P.K.A.); sandhari@augusta.edu (S.A.); jfarmaha@augusta.edu (J.F.); jawoodall@augusta.edu (J.W.)

**Keywords:** next-generation sequencing (NGS), comprehensive gene panel (CGP), solid tumor genomic profiling, analytical validation, clinical utility

## Abstract

**Background:** Comprehensive genomic profiling (CGP) via next-generation sequencing (NGS) is pivotal for precision oncology, yet many laboratories face challenges with incomplete genomic coverage, complex bioinformatics workflows, and limited integration of key biomarkers. **Methods**: We evaluated the analytical performance and clinical utility of a CGP assay using 119 tumor samples representing 18 types of cancer, previously analyzed with an orthogonal NGS panel. Concordance was assessed across 81 genes, covering 176 single-nucleotide variants (SNVs), eight copy number variations (CNVs), four deletions, one duplication, and four gene fusions. Limit of detection (LOD) studies employed AcroMetrix Mutant Hotspot Control and SeraSeq Lung and Brain CNV Mix. Microsatellite instability (MSI) and tumor mutational burden (TMB) were quantified. Inter- and intra-run reproducibility were evaluated to assess precision. **Results**: The CGP assay demonstrated high analytical performance, with >99% sensitivity, 100% specificity, and complete accuracy for variant detection. LOD studies revealed robust detection of SNVs at ≤5% variant allele frequencies (VAF) and CNVs at three copies. MSI and TMB results were consistent with clinical expectations, showing minimal bias compared to the orthogonal panel. Inter- and intra-run testing confirmed 100% reproducibility, indicating strong assay precision. Post-sequencing variant reporting was streamlined using the iCare platform, enabling direct FASTQ-to-report generation without intermediate bioinformatic steps. **Conclusions**: These findings support the present assay’s clinical utility in personalized oncology assessment.

## 1. Introduction

Numerous tumor-specific variants underpin critical diagnostic, prognostic, and therapeutic decisions across cancer types, driving the shift toward personalized oncology [1]. Next-generation sequencing (NGS) has emerged as the preferred method for molecular analysis, enabling efficient, multiplexed variant detection across diverse tumor types [2]. However, many laboratories rely on small DNA-based panels (typically 50–100 genes), which, while cost-effective and rapid, yield incomplete mutation profiles [3]. These limited panels often fail to comprehensively screen known hotspots and struggle to reliably assess pan-tumor biomarkers like microsatellite instability (MSI) and tumor mutational burden (TMB), compromising their utility in capturing the full genomic landscape essential for precision medicine [4,5].

NGS gene panels with a high number of target genes, such as those encompassing 500 or more genes (such as Tempus xT (648 genes), TruSight Oncology 500 (523 genes), and MSK-IMPACT (468 genes), etc.) have become increasingly vital in cancer diagnostic settings [6,7,8]. These comprehensive panels enable the simultaneous analysis of a vast array of genetic alterations such as single-nucleotide variants (SNVs), insertions and deletions (indels), copy number variations (CNVs), and structural rearrangements across numerous genes implicated in disease pathology [1]. This broad scope is critical for effective tumor management, as it allows clinicians to identify actionable molecular anomalies that can guide personalized treatment strategies, such as targeted therapies or immunotherapies [9]. Such large panels can uncover rare or low-frequency variants that might be missed by smaller, more focused panels, providing a more complete genomic profile of a patient’s tumor [3].

However, despite the existence of numerous targeted gene panels, the continuous development of new panels is driven by the dynamic nature of genomic research and clinical needs. Advances in cancer biology and molecular diagnostics regularly reveal new gene variants associated with tumorigenesis, drug response, or resistance, necessitating the addition of these targets to existing panels [10]. Conversely, some genes previously included in panels may be deprioritized as their clinical relevance diminishes based on emerging evidence [1]. The discovery of novel oncogenic drivers or resistance mutations, often identified through large-scale genomic studies or real-world clinical data, prompts the design of updated panels to ensure they remain relevant [11]. Additionally, improvements in sequencing technology, bioinformatics pipelines, and assay chemistry enable the creation of more robust and sensitive panels that can detect variants at lower allele frequencies or with greater reproducibility, addressing limitations of earlier iterations [12]. This iterative process reflects the need to adapt to the evolving landscape of precision medicine, where staying ahead of the curve in identifying all actionable variants is paramount for patient care [13].

Furthermore, beyond designing comprehensive panels to identify relevant mutations, it is equally critical to address the post-sequencing analysis workflow, ensuring that technologists can efficiently process the large datasets generated by these expansive panels. A significant challenge in molecular diagnostic labs is the complexity and time-intensive nature of handling sequencing data, particularly when turnaround times are constrained by urgent patient conditions [14]. Current workflows often involve multiple steps: raw sequencing data in FASTQ format must be demultiplexed, converted into aligned BAM files, and processed to generate variant call format (VCF) files, which are then fed into third-party bioinformatics pipelines or annotation tools to produce clinically meaningful variant reports [15]. This fragmented process is not only labor-intensive but also prone to errors, requiring skilled bioinformaticians to oversee each stage, which can overwhelm diagnostic labs with limited resources [16]. Molecular diagnostic labs urgently need a one-stop solution that streamlines this process, allowing technologists to input a single type of post-sequencing file (e.g., FASTQ) and receive fully annotated variant reports without navigating a maze of intermediate steps and tools [17]. Such integrated platforms exist in limited forms, such as commercial solutions like Illumina’s DRAGEN or Thermo Fisher’s Ion Reporter, which aim to consolidate analysis, but they are often tied to specific sequencing hardware or lack the flexibility to handle custom panels [15].

Moreover, these solutions may not fully address the annotation and prioritization of variants in a clinically actionable context, leaving labs reliant on additional third-party tools like OncoKB, VarSome, or Qiagen’s cloud connect [16,17]. The absence of a user-friendly pipeline exacerbates disparities in diagnostic capabilities, particularly for smaller labs, and hinders the scalability of NGS in routine clinical practice [18]. Developing such a streamlined, end-to-end analysis framework is not just a technical convenience but a necessity to maximize the impact of high-target gene panels, ensuring that the promise of precision oncology is not bottlenecked by bioinformatics inefficiencies.

The novelty of the present study resides in the validation of a high-content 1080-gene CGP assay integrated with a cloud-based bioinformatics platform that accepts raw FASTQ files and delivers fully annotated clinical reports without intermediate manual steps, an approach distinct from existing solutions tied to specific hardware or requiring dedicated bioinformatics expertise. To this end, we describe the extensive validation of OncoIndx (1Cell.Ai, Foster City, CA, USA), a targeted NGS assay designed to detect SNVs, CNVs, indels, gene fusions, MSI, and TMB from a single DNA input across 1080 genes. The validation utilized a diverse cohort of 140 solid tumor samples, including patient-derived FFPE specimens, College of American Pathologists (CAP) proficiency testing material, and characterized reference standards, sequenced on the Illumina NextSeq 550 Dx (Illumina, San Diego, CA) and benchmarked against the TruSight Oncology 500 (TSO-500) panel. The analysis demonstrated concordant results across all variant classes, achieving >99% sensitivity, 100% specificity, precision, and accuracy. This integrated single-assay approach enhances efficiencies in sample utilization, turnaround time, and cost compared to multi-assay workflows, supporting the adoption of OncoIndx and iCare (1Cell.Ai, Foster City, CA, USA) in clinical settings for comprehensive solid tumor genomic profiling.

## 2. Materials and Methods

### 2.1. Sample Information

This retrospective study adhered to the Association for Molecular Pathology (AMP) and College of American Pathologists (CAP) guidelines for clinical assay validation, in line with the Declaration of Helsinki. The study was approved by the Institutional Review Board (IRB #00000150, HAC IRB #611298) at Augusta University, Augusta GA, USA, with all protected health information (PHI) removed and data anonymized prior to analysis. The study included 107 patient samples from 18 tumor types, sourced from banked DNA of formalin-fixed, paraffin-embedded (FFPE) specimens (Table S1). A total of 12 CAP proficiency testing (CAP-PT) samples with known single-nucleotide variants (SNVs) were also analyzed. Patient samples were analyzed in duplicate for inter- and intra-run consistency across different runs. Reference controls comprised AcroMetrix Oncology Hotspot Control (#969056, Thermo Scientific, Fremont, CA, USA) with over 500 COSMIC database mutations across 53 genes (evaluated for 14 genes, 17 variants; Table S2) and Seraseq Lung & Brain CNV Mix (+12 copies, #0710-0416, SeraCare Life Sciences, Milford, MA, USA) assessing *EGFR*, *MYCN*, and *MET* copy number variations (CNVs). All samples were previously sequenced on the NextSeq 550 DX using the orthogonal CGP assay, with data retrieved from the repository for analysis.

### 2.2. Sample Processing and Quality Assessment

Tumor content in patient samples was assessed and annotated by a board-certified pathologist using hematoxylin and eosin-stained tissue sections, with a 20% tumor content threshold established for analysis. Macrodissection was performed on two to five unstained 5 µM sections, followed by deparaffinization and DNA extraction using the QIAamp DNA FFPE Tissue Kit (#56404, QIAGEN, Germantown, MD, USA) per manufacturer’s instructions. DNA quality was evaluated with a Nanodrop spectrophotometer (Thermo Fisher Scientific, Waltham, MA, USA), accepting an OD 260/280 ratio of 1.7–2.2. The dsDNA concentration was measured using the Qubit 1X High Sensitivity dsDNA Kit (#Q33231, Thermo Fisher Scientific, Waltham, MA, USA). Samples passing quality control were diluted with RNase/DNase-free water to a target concentration, optimized for a 100 ng dsDNA input in the assay.

### 2.3. Sample Dilution for Analytical Analysis (Limit of Detection Study)

For limit of detection (LOD) studies, CNVs and SNVs were analyzed using validated reference materials with serial sample dilutions. SNV LOD was assessed with AcroMetrix Oncology Hotspot Control (VAF 5–15%), tested undiluted (100%, 100 ng) and diluted to 50% (50 ng), 25% (25 ng), 10% (10 ng), and 5% (5 ng) with wild-type DNA to determine the minimum detectable variant allele frequency (VAF). CNV LOD was evaluated using Seracare Lung and Brain CNV Mix, undiluted (100%, 12 copies) and diluted to 50% (6 copies) and 25% (3 copies) with wild-type DNA. Wild-type genomic DNA used for dilution studies (to maintain a consistent 100% final input across all dilutions) was obtained from peripheral blood samples of individuals with no known malignancy and met the same laboratory quality control criteria as clinical specimens, including A260/280 ratios between 1.8 and 2.0.

### 2.4. OncoIndx Library Preparation and Sequencing

**Figure 1:**
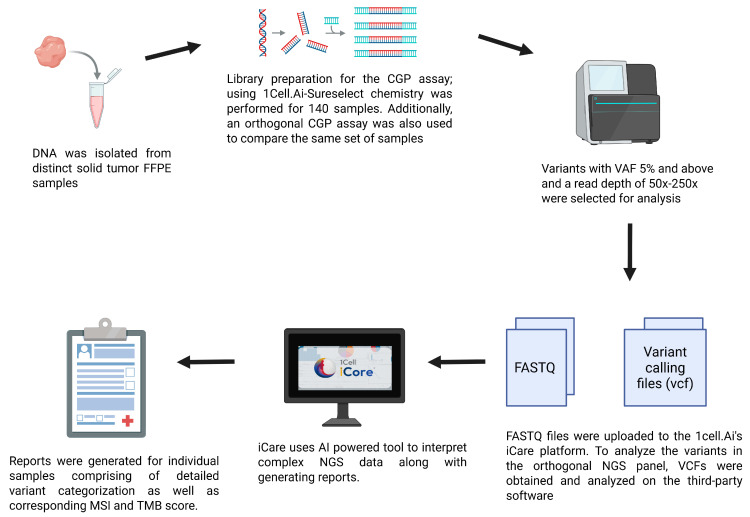
Schematic representation of the complete workflow, from library preparation and sequencing to post-sequencing analysis and final variant report generation. (Created in BioRender. VASHISHT, A. (2026) https://BioRender.com/w2uk8f3).

Library preparation was conducted in accordance with the manufacturer’s protocol using the SureSelect XTHS2 DNA Library Preparation Kit (Agilent, Santa Clara, CA, USA). Genomic DNA (gDNA) was enzymatically fragmented utilizing the SureSelect Enzymatic Fragmentation Kit (Agilent, Santa Clara, CA, USA), followed by end repair, A-tailing, and adapter ligation. Adapter-ligated gDNA fragments were subsequently amplified using SureSelect XT HS2 Index Primer Pairs for ILM (Pre-PCR, Index Pairs 1–96). Target enrichment was achieved through hybridization with a custom-designed SureSelect probe (Tier-2, 0.5–2.9 Mb) tailored for the OncoIndx 1080-gene panel (Table S3), followed by capture, PCR amplification, and subsequent cleanup. The resulting PCR product was quantified using the Qubit 1X High Sensitivity dsDNA Kit (Q33231, Thermo Fisher Scientific, Waltham, MA, USA). The fragment size distribution was assessed on the 4150 TapeStation system using High Sensitivity D1000 ScreenTapes (Agilent, Santa Clara, CA, USA). Libraries were normalized based on fragment size, pooled at 4 nM, and adjusted to a final loading concentration of 1.4 pM. Sequencing was conducted with 20 samples per run using the NextSeq 550 Dx (Illumina, San Diego, CA, USA) in Research Use Only (RUO) mode with a V2 flow cell kit (Illumina, San Diego, CA, USA). A 151 bp paired-end configuration with 8-base pair indexes was employed over 316 cycles. Raw sequencing reads were converted to FASTQ format via the BaseSpace TSO 500 Assessment App (Illumina, San Diego, CA, USA) and subsequently uploaded to the iCare platform for variant calling. The variants were analyzed for concordance with the prior established CGP assay, which used the QIAGEN Clinical Insight Interpret (QCI-I) as the variant calling tool. The complete workflow of the assay is presented in Figure 1.

### 2.5. iCare Variant Calling and CNV Analysis

Variant detection was executed using the proprietary iCare™ software platform (1Cell.Ai), leveraging computational algorithms comparable to the Broad Institute’s BWA-GATK HaplotypeCaller Best Practices Workflow. Reads were aligned to the human reference genome GRCh38/hg38. The platform integrates multiple cancer-associated databases, including ClinVar (NCBI) and a curated proprietary database within iCare™, supplemented by in silico prediction tools (SIFT and PolyPhen) to enhance variant prioritization and determine clinical significance. Default variant selection criteria were established as follows: Variant Allele Frequency (VAF) ≥ 0.2% for tissue samples, total sequencing depth ≥ 100 reads, alternate allele counts of ≥5 for hotspot variants, ≥10 for non-hotspot variants, and ≥10 for variants of unknown significance, with a PHRED quality score threshold of 30. Although the iCare platform supports variant detection at a default VAF ≥ 0.2%, the threshold was adjusted to ≥5% for this study to match the filtering criteria of the orthogonal TSO-500/QCI-Interpret pipeline, ensuring a methodologically equivalent concordance comparison. The configurable VAF threshold within iCare allows laboratories to tailor analytical sensitivity to their specific clinical requirements. CNV analysis employed a dual-algorithm approach: CNVkit for genome-wide segmental detection using a pooled panel-of-normals reference, and PureCN for focal amplification/deletion refinement with concurrent tumor purity and ploidy estimation, both aligned with Best Practices Workflow standards. Fusion transcript analysis was conducted using FuSeqWES, which predicts gene fusions from alignment (BAM) files, employing computational techniques consistent with established Best Practices Workflow methodologies.

### 2.6. MSI and TMB Analysis Using iCare

MSI status was determined using MSIsensor2, which generates a continuous score representing the proportion of unstable microsatellite loci across the genome. For clinical reporting within the iCare workflow, scores were categorized as microsatellite stable (MSS; score = 0), microsatellite instability-low (MSI-L; score 1–19), and microsatellite instability-high (MSI-H; score ≥ 20). TMB was assessed using the maftools R package, which processes Mutation Annotation Format (MAF) files. TMB was categorized as TMB-High (≥10) or TMB-Low (<10). Variants with a quality score below 20 were excluded from analysis, and only variants with pathogenic clinical significance were considered in the final TMB assessment.

### 2.7. Library Preparation and Sequencing Protocol for the Orthogonal CGP Assay

Samples that passed quality control (QC) were processed for library preparation using the TSO-500 hybrid capture-based kit (#20028214, Illumina, San Diego, CA, USA) according to the manufacturer’s protocol. The gDNA was first fragmented to an average size of approximately 130 bp using an ultrasonicator (Covaris, Woburn, MA, USA). In parallel, RNA was converted to complementary DNA (cDNA) using the cDNA synthesis module included in the TSO-500 kit. Both Covaris-processed gDNA and cDNA underwent end repair, A-tailing, and adapter ligation, followed by index PCR amplification (UP-index).

Library enrichment was performed via hybrid capture utilizing probes specific to 523 genes of interest, including 54 gene fusions. This was followed by PCR-based enrichment, cleanup, and quantification of dsDNA using the Qubit 1X High Sensitivity dsDNA Kit (Q33231, Thermo Fisher Scientific, Waltham, MA, USA). The libraries were normalized through bead-based methods before sequencing. Sequencing was conducted on the NextSeq 550 DX platform (Illumina, San Diego, CA, USA) using V2 sequencing reagent kits, in full compliance with the manufacturer’s guidelines.

### 2.8. Post Sequencing Data Analysis for the Orthogonal CGP Assay

The raw sequence reads in FASTQ format were converted to BAM and VCF files using Qiagen Cloud Connect (QCC) clinical decision support software (Qiagen, Germantown, MD, USA). MSI and TMB information was extracted directly from the VCF files. These VCF files were subsequently uploaded to Qiagen Clinical Insight Interpret (QCI-I) (Qiagen, Germantown, MD, USA) for the analysis of single-nucleotide variants (SNVs), copy number variations (CNVs), and indels/duplications. Variants were filtered for analysis based on a variant allele frequency (VAF) greater than 5% and a total read depth exceeding 250×. The variants were then classified into tiers using evidence-based literature and manually curated data within the Qiagen Clinical Insight interpreter (QCI-I). The RNA data were analyzed using Illumina’s TST170 BaseSpace App, with high-confidence variant data exported as .csv files. All detected alterations were reported in accordance with the joint consensus recommendations of the Association for Molecular Pathology (AMP), American Society of Clinical Oncology (ASCO), and College of American Pathologists (CAP).

### 2.9. Performance Metric Evaluation

The performance metric was calculated for both clinical and reference control samples for SNVs, CNVs, gene fusions, and indels. Seven performance criteria were assessed, including positive percentage agreement (PPA), negative percentage agreement (NPA), positive predictive value (PPV), negative predictive value (NPV), accuracy, false-negative rate (FNR), and false-positive rate (FPR).

### 2.10. Statistical Analysis

The data obtained were recorded in Microsoft Excel and analyzed using GraphPad Prism 8 (version 8.0.2, San Diego, CA, USA). A Bland–Altman analysis was conducted to assess the agreement between the TSO-500 and OncoIndx chemistries. This analysis involved plotting the difference in the number of samples where TMB and MSI were detected by each assay against the average number of samples in which these parameters were detected by both assays.

## 3. Results

### 3.1. Patient Demographics and Quality Control Measures

In the present study, a final cohort of 107 patients was established, consisting of 89 females (median age: 65 years) and 18 males (median age: 72 years), following the incorporation of additional samples. A total of 140 samples were successfully processed across seven sequencing runs, with no run failures recorded. From an initial pool of patient samples, quality control revealed that 50 samples failed to achieve the requisite tumor cellularity of ≥20%, while 20 FFPE-derived DNA samples were excluded due to suboptimal integrity. Additionally, 13 samples yielded DNA concentrations below the assay’s minimum threshold, and 10 exhibited heterogeneous tumor cellularity across FFPE sections despite an average exceeding 20%, rendering them unsuitable for reliable analysis. Targeted variant detection across these tumor types demonstrated 100% concordance with expected profiles, affirming the assay’s consistency and reliability across a diverse solid tumor spectrum.

### 3.2. Genomic Variant Detection and Analytical Performance of OncoIndx

**Figure 2:**
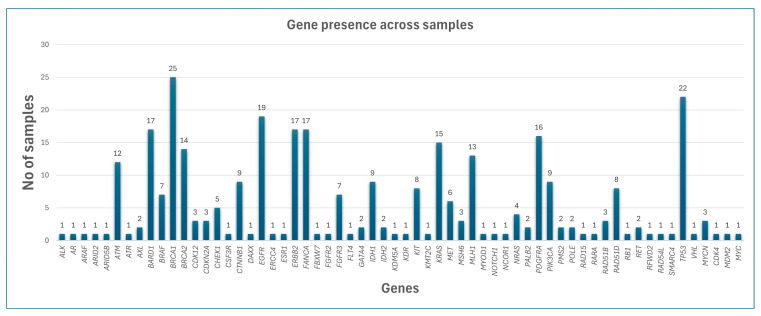
Prevalence of genetic alterations in the analyzed cohort. Bar graph depicting the number of samples harboring alterations in each of the 81 genes interrogated across 140 solid tumor specimens. The majority of genes displayed alterations in 1–3 samples, underscoring the heterogeneous mutational landscape characteristic of solid tumor malignancies. Notably, *BRCA1*, *TP53*, *EGFR*, *ERBB2*, *FANCA*, *BARD1*, *PDGFRA*, and *KRAS* emerged as the most frequently altered genes within this cohort. Genes subjected to dual interrogation for both SNVs and CNVs, including *EGFR* and *ERBB2*, are represented accordingly.

In this investigation, a comprehensive dataset of 81 genes was interrogated, targeting 176 single SNVs, eight CNVs, four deletions, one duplication, and four gene fusions (Table S4). Across the samples analyzed with the CGP assay, all 81 genes were successfully identified. Of the 176 SNVs that were analyzed on TSO-500, 175 were detected with high fidelity, with the sole exception being *BRCA2* p.V1862fs, which was not detected. All eight CNVs, four deletions (notably *EGFR* p.E746_A750del), and one duplication (*ERBB2* p.Y772_A775dup) were accurately identified. Among the 81 genes, *BRCA1*, *TP53*, *EGFR*, *ERBB2*, *FANCA*, *BARD1*, *PGDFRA*, and *KRAS* emerged as the most frequently detected, with *EGFR* and *ERBB2* subjected to dual interrogation for both SNVs and CNVs (Figure 2). To evaluate the clinical utility of the assay, analytical performance metrics, including clinical sensitivity (PPA), specificity (NPA), precision (PPV), NPV, FNR, and FPR, were calculated. These metrics, aggregated across variant classes, are presented in Table 1, demonstrating robust performance across a heterogeneous genomic profile.

### 3.3. Gene Fusion Profiling

To evaluate the performance of the panel’s gene fusion assay, five samples were analyzed for previously identified fusions (*NCOA4*-*RET*, *ST7*-*MET*, *ALK-TNS1*, *ALK-EML*4) detected by orthogonal CGP assay (Table 1). All specified fusions were confirmed in the five samples, achieving 100% concordance, with *NCOA4-RET* detected in two samples. Additionally, the OncoIndx gene panel was applied to 107 samples, revealing significant gene fusions involving therapeutically relevant genes. Table 2 summarizes the number of fusions, their partner genes, and the number of unique samples harboring these fusions for key oncogenic drivers.

### 3.4. Determination of Limit of Detection (LOD)

To assess the limit of detection (LOD) for the assay’s sensitivity in identifying variant calls, two reference materials, Acrometrix Mutant Hotspot Control (for SNVs) and SeraSeq Lung and Brain CNV Mix, were utilized. For SNVs, the Acrometrix control was further diluted into four concentrations, 50%, 25%, 10%, and 5%, as outlined in the methods section. For the genes *NRAS*, *ALK*, *CTNNB1*, *PIK3CA*, *PDGFRA*, *KIT*, *FGFR2*, *KRAS*, *AKT1*, *TP53*, and *GNAS*, the target VAF ranged from 5% to 15%, while for *EGFR*, *MET*, and *BRAF*, the target VAF ranged from 15% to 35%, as specified by the supplier’s instructions. Here, a 100% DNA input corresponds to the undiluted control with a VAF of 5–15%.

The assay successfully detected all 14 genes and 21 associated variants at the 100% and 50% VAF input levels. At lower dilutions, most variants were still detected across all concentrations, with a few exceptions: the *ALK* p.F1174L variant was not detected at the 5% dilution; the *PIK3CA* variants p.E542K and p.E545K were only detected at 100% and 50% inputs; the *PIK3CA* p.H1047R variant was not detected at 5%; and the *EGFR* p.E746_A750delELREA variant was also undetectable at the 5% dilution (Table S5). This evaluation demonstrates that while the OncoIndx assay exhibits robust sensitivity across a range of dilutions for most variants, its analysis of specific variants at the lowest (5%) concentration defines the practical LOD for those targets.

**Figure 3:**
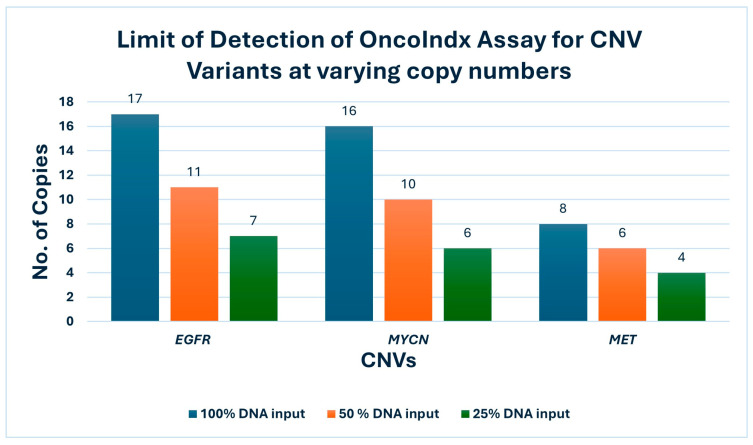
Bar graphs showing the observed CNVs for *EGFR*, *MYCN*, and *MET* genes using the OncoIndx assay at different copy number dilutions.

Similarly, for CNV, SeraSeq, *EGFR*, *MYCN*, and *MET* copy numbers were analyzed for LOD. Likewise, the Seseq material was diluted into 50% (minimum six copies) and 25% (3 copies). Seraseq (100%) contained undiluted material with a minimum of 12 copies each of the three genes. All the CNVs were detected in all the concentrations (Figure 3).

### 3.5. MSI and TMB Concordance Between OncoIndx and TSO500

TMB and MSI were successfully detected across all samples in the OncoIndx analysis. Among the 107 samples analyzed (excluding the CAP, Acrometrix, and SeraSeq control materials), the distribution of samples with MSI status was as follows: 58 MSI-Stable, 43 MSI-Low, and six MSI-High. In terms of TMB classification, 66 samples were classified as TMB-High (≥10 mutations per megabase), while 41 samples were classified as TMB-Low (<10 mutations per megabase).

**Figure 4:**
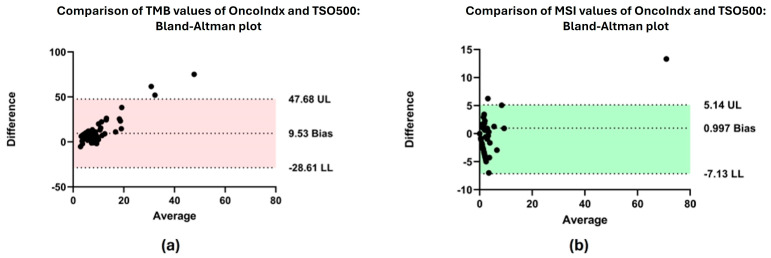
Bland–Altman plots were used to evaluate agreement between the OncoIndx and TSO500 assays by plotting the differences between paired measurements against their mean. (**a**) For TMB estimation, a bias of 9.53 was observed. Individual black dots represent paired differences for identical mutations measured by both assays. (**b**) For MSI detection, the analysis showed a negligible bias of −0.997, indicating minimal systematic difference between the two methods. The solid central line denotes zero bias, while the dotted lines indicate the mean bias, reflecting any consistent over- or underestimation by one method relative to the other. The concentration of data points around the zero-difference line demonstrates strong overall agreement between the assays. The upper (UL) and lower (LL) limits of agreement, shown as the shaded region, represent the range within which 95% of the differences between the two methods are expected to fall.

To evaluate the comparative performance of the two assays, 65 samples were analyzed using both OncoIndx and TSO500, and the results were compared through Bland–Altman analysis, as depicted in Figure 4. For MSI detection, the analysis revealed a negligible bias of −0.997, with upper and lower limits of agreement at 5.14 and −7.13, respectively. In contrast, the Bland–Altman analysis for TMB estimation revealed a bias of 9.53, with upper and lower limits of agreement at 47.68 and −28.61, respectively.

### 3.6. Inter- and Intra-Run Reproducibility

A total of seven distinct samples were subjected to both inter-run and intra-run analyses to evaluate the reproducibility and analytical robustness of the CGP assay. In the inter-run assay, designed to assess consistency across independent sequencing runs, samples 8F-SP22-8769-B1 and HRD1_SP20-12588-B5 were processed across three separate runs, while the remaining five samples—namely 16_TA5_SP22-7596-B1, 17_TA6_SP22-15835-A7, 18_TA12_SP20-475-A7, 19_TA16_SP22-6870-B1, and 20_TA18_SP22-6326-A1—were evaluated in two distinct runs (Table S6). Conversely, the intra-run analysis, aimed at verifying precision within a single run, utilized only samples 8F-SP22-8769-B1 and HRD1_SP20-12588-B5, each tested in triplicate to ensure reliability (Table S7). Across all seven samples, the expected variants encompassing a range of clinically relevant genetic alterations such as *KRAS* p.Q61H, *CTNNB1* p.T41A, and *BRCA1* p.E23Vfs*17, among others, were consistently detected with 100% concordance between expected and observed results. This alignment underscores the high reproducibility and fidelity of the OncoIndx gene panel in identifying targeted genomic variants across both inter- and intra-run conditions.

## 4. Discussion

The swift integration of NGS platforms into clinical diagnostic laboratories has fundamentally transformed genetic testing paradigms, heralding an era of unprecedented opportunity to characterize an extensive array of actionable driver genes within tumor specimens [19,20,21,22]. A hallmark advantage of NGS-based CGP somatic panels lies in their efficiency: requiring merely 80–120 ng of DNA, these panels yield a wealth of clinically actionable insights, encompassing SNVs, CNVs, MSI, TMB, and gene fusions, thereby facilitating tailored therapeutic decision-making with minimal tissue input. For patients with solid tumors, the National Comprehensive Cancer Network (NCCN) guidelines advocate the deployment of multiplex gene panels and NGS-based analyses to achieve a holistic prognostic assessment, underscoring the critical role of broad molecular profiling in identifying therapeutically relevant genomic alterations [23]. This recommendation is complemented by seminal reports, including those from The Cancer Genome Atlas (TCGA), which delineates a complex network of genetic perturbations characterized by a paucity of universally recurrent mutations across solid malignancies [24,25]. Among the recurrent alterations elucidated in solid tumors are genes orchestrating epigenetic regulation (e.g., *ARID2*, *KMT2C*), tumor suppression (e.g., *BRCA1*, *BRCA2*, *ATM*, *MLH1*), oncogenic activation (e.g., *ALK*, *BRAF*, *KRAS*, *PIK3CA*, *EGFR*), and transcriptional or differentiation processes (e.g., *CTNNB1*, *NOTCH1*, *DAXX*) [26,27,28,29,30,31,32,33,34]. These findings underscore the heterogeneity of solid tumor genomes, with an ever-expanding catalog of altered loci that continues to evolve as genomic datasets gather.

Despite the transformative potential of NGS CGP, many clinical laboratories persist in employing panels with restricted genomic coverage, typically encompassing approximately 54 genes, which fall short of capturing the full mutational landscape [35]. Such limited panels frequently omit critical hotspots and emerging driver genes, thereby constraining the delineation of a comprehensive mutation profile and impeding the realization of fully personalized diagnostic and therapeutic strategies. In contrast, broader panels, such as those exceeding 500–1000 genes, enable a more exhaustive interrogation of the genomic milieu.

To this end, we rigorously evaluated the clinical performance of the OncoIndx panel, a 1080-gene NGS-based assay, by screening 140 solid tumor specimens encompassing 18 distinct histological types. Analytical performance assessments underscored the panel’s operational simplicity and clinical utility, with library preparation requiring approximately 3 h of hands-on time (exclusive of PCR amplification and incubation periods), followed by a sequencing duration of approximately 36 h for 20 samples on a NextSeq V2 flow cell. Sequencing run parameters consistently exceeded the manufacturer’s recommended thresholds, exhibiting exceptional uniformity across samples for each metric, both within individual runs and across multiple runs, as evidenced by robust inter- and intra-run reproducibility. The capacity to simultaneously sequence 20 solid tumor samples in a single run confers substantial advantages to clinical laboratories, optimizing cost-effectiveness, temporal efficiency, and throughput.

Furthermore, the assay exhibited an exceptional sensitivity of 99.43%, specificity of 100%, and accuracy for SNVs, as well as 100% sensitivity, specificity, and accuracy for CNVs, indels, duplication, and gene fusions. LOD experiments utilizing the AcroMetrix reference material demonstrated that, as anticipated, assay sensitivity decreased with increasing dilution. Nonetheless, the assay maintained an impressive detection capability, accurately identifying 76% of variants even at the lowest VAF of approximately 5%. Moreover, the platform consistently detected CNVs across all tested copy number levels with 100% accuracy.

Beyond analytical performance evaluation, the clinical utility of comprehensive genomic analysis in solid tumors was also assessed. The inherent molecular heterogeneity of the tumors was evident, with 176 SNVs and eight CNVs identified across 81 genes. Comparable results were reported by Hartmaier et al. in a study employing the FoundationOne 405-gene panel, wherein 81 solid tumor samples were sequenced [13]. However, the orthogonal validation performed in that study covered only 20% of the samples (16/81) and was conducted using a smaller orthogonal panel. This limited validation scope and panel size reduce confidence in the dataset, particularly for genes lacking orthogonal confirmation.

Complementing these findings, the OncoIndx gene fusion panel was evaluated in five samples harboring previously identified fusions (*NCOA4-RET*, *ST7-MET*, *ALK-TNS1*, *ALK-EML4*) detected by the TSO500 gene panel, achieving 100% concordance. Extending this to 107 samples, the panel revealed a diverse array of clinically significant gene fusions involving key oncogenic drivers. Notably, *ROS1* demonstrated the highest prevalence with 22 fusions across 19 samples, involving partners such as *CSMD1*, *EYA1*, and *PRKD1*, consistent with its established role as a recurrent driver in non-small cell lung cancer and other solid tumors, where fusion frequencies range from 0.47% to 1.6% in pan-tumor analyses [36,37]. *NTRK2* followed with 12 fusions in 10 samples, encompassing partners like *RGS6* and *PPARG*, aligning with the low but actionable incidence of *NTRK* fusions that confer sensitivity to targeted inhibitors such as entrectinib and larotrectinib [38,39]. Other fusions, including those in *ALK* (e.g., with *EML4* and *TNS1* in 3 samples), *MET*, *NTRK1*, and *NTRK3*, were detected at lower frequencies (2–4 fusions across 2–4 samples), underscoring their prognostic and therapeutic implications in subsets of lung, colorectal, and other malignancies [40,41,42,43]. Rare events involving *PAX3*, *PAX7*, *RET*, *SS18*, and *YAP1* in this study were each confined to single samples, highlighting the panel’s capacity to uncover low-frequency yet potentially actionable alterations that may guide precision therapies like selpercatinib for *RET* fusions [44]. This fusion profiling enhances the panel’s utility in capturing the full spectrum of genomic drivers, addressing the heterogeneity of solid tumors, and supporting recent recommendations for routine NGS-based fusion detection in advanced cancers to optimize patient stratification and targeted interventions.

TMB serves as a critical biomarker in solid tumors, reflecting the genomic instability and potential immunogenicity of malignancy. Several studies utilizing NGS panels have reported TMB values in cohorts of 50–100 solid tumor samples, providing a comparative framework for assessing mutational load. Hartmaier et al. (2017) in their 81 solid tumor samples (breast, lung, colorectal) reported a median TMB of six mutations per megabase (m/Mb), with subtype averages ranging from 6.3 m/Mb (breast) to 10.8 m/Mb (colorectal) [45]. Similarly, Zehir et al. (2017) examined a subset of 62 advanced solid tumors using the MSK-IMPACT 410-gene panel, documenting a median TMB of 7.2 m/Mb, with lung cancers averaging approximately 7.2 m/Mb and breast cancers at 5–6 m/Mb [11]. Frampton et al. (2016) validated the FoundationOne panel (315–405 genes) in 73 solid tumor samples, yielding a median TMB of 9 m/Mb [3], while Chalmers et al. (2017) reported median TMB values of 9.0 m/Mb (67 lung adenocarcinomas) and 10.8 m/Mb (92 colorectal cancers) in tumor-specific subsets of 50–100 samples [4].

In contrast, our study showed an average TMB of 15 m/Mb across 107 samples with available data. This value substantially exceeds the TMB reported in the aforementioned studies (6–10.8 m/Mb), suggesting a cohort enriched with high-TMB tumor types, such as melanoma, lung, and ovarian cancers, or MSI-H cases, which often exhibit TMB values above 10–20 m/Mb. The broader 1080-gene panel, compared to the 400–500-gene panels in prior studies, may also enhance mutation detection, though TMB normalization per megabase mitigates this effect. The lower TMB values in Hartmaier et al., Zehir et al., Frampton et al., and Chalmers et al. likely reflect more diverse or less mutagen-exposed cohorts, with fewer hypermutated outliers. These comparisons underscore the variability of TMB across solid tumors and highlight the potential of larger panels to capture elevated mutational loads in clinically distinct populations.

Furthermore, for MSI detection, the Bland–Altman analysis revealed a negligible bias of 0.997, with upper and lower limits of agreement at 5.14 and −7.13, respectively, indicating a minor systematic difference wherein 1Cell.Ai’s CGP assay tends to yield slightly higher MSI values compared to TSO500. This suggests that the two assays demonstrate good agreement for MSI detection, characterized by minor bias and relatively narrow limits of agreement. In contrast, the Bland–Altman analysis for TMB estimation revealed a bias of 9.53, with upper and lower limits of agreement at 47.68 and −28.61, respectively. This bias indicated that OncoIndx tends to overestimate TMB values compared to TSO-500. The results suggested that assays generally identify TMB-High and TMB-Low categories consistently; quantitative discrepancies exist, especially at higher TMB values.

While a systematic bias in TMB estimation was observed, it is important to note that TMB is typically applied as a categorical biomarker in clinical settings (e.g., TMB-High versus TMB-Low). In this study, both assays demonstrated concordant classification across these clinically relevant categories, suggesting that the observed bias may have limited impact on clinical interpretation.

However, the presence of quantitative differences indicates that absolute TMB values are not directly interchangeable between platforms. This highlights the potential need for platform-specific thresholds or calibration approaches, particularly for cases with higher TMB values, and represents an important area for future investigation.

The iCare platform provides an efficient and user-friendly solution for post-sequencing variant analysis, streamlining workflows that are typically fragmented across multiple steps in conventional pipelines. Standard analysis approaches often require separate preparation of sample sheets for BCL conversion and downstream variant calling, along with multiple manual data-handling steps. These processes can increase the risk of user-related errors and add complexity to the workflow.

In contrast, the iCare platform simplifies post-sequencing analysis by enabling direct processing of sequencing data within a unified framework, minimizing manual intervention and reducing workflow complexity. This streamlined approach facilitates more efficient data handling and interpretation in clinical settings.

The analytical performance of the platform was validated through comparison with an orthogonal, clinically validated assay, demonstrating high concordance in variant detection. While detailed time-based benchmarking was not performed in this study, the reduced need for manual processing steps highlights the potential of iCare to improve overall workflow efficiency in genomic data analysis.

Thus, OncoIndx’s exceptional sensitivity, specificity, and comprehensive genomic profiling capabilities position it as a powerful tool for personalized oncology care. Its ability to efficiently and accurately detect a wide range of genomic alterations makes it highly suitable for clinical applications, offering significant potential to guide therapeutic decision-making, monitor disease progression, and optimize patient outcomes in the clinical setting. The entire workflow’s rapid turnaround time further enhances its utility in clinical diagnostics, enabling timely interventions and improving overall patient management.

## 5. Limitations

Several limitations of the present study warrant acknowledgment. The retrospective cohort, while encompassing 18 tumor types, is numerically weighted toward ovarian and breast malignancies, reflecting institutional sample availability rather than prospective design. A larger, demographically and histologically balanced multi-tumor cohort would further substantiate the assay’s analytical performance across underrepresented tumor types. Another limitation of this study is that a fully defined true-negative cohort across the entire 1080-gene panel was not available; therefore, specificity and related metrics could not be robustly estimated and should be interpreted with caution.

Also, samples below 20% tumor cellularity were excluded from analysis, which may not reflect real-world low-cellularity specimens routinely encountered in clinical practice. Furthermore, gene fusion detection was performed using a DNA-based algorithm (FuSeqWES); RNA-based orthogonal validation would further strengthen fusion calling confidence, as RNA sequencing remains the gold standard for fusion confirmation.

Another limitation of this study is the lack of LOD evaluation for gene fusions and small indels/duplications due to the limited availability of well-characterized samples for these variant classes, which will be addressed in future studies.

## 6. Conclusions

In conclusion, the OncoIndx 1,080-gene CGP assay demonstrated robust analytical performance across all variant classes, achieving >99% sensitivity and 100% specificity in a diverse solid tumor cohort. Its integration with the iCare cloud-based platform streamlines post-sequencing analysis, offering a scalable and clinically deployable solution for comprehensive genomic profiling in precision oncology.

## Figures and Tables

**Table 1:** Diagnostic precision and concordance of OncoIndx across genomic variant classes.

Variant Type	Total Variants	Detected	Sensitivity (PPA) %	Specificity (NPA) %	Precision (PPV) %	NPV %	FNR %	FPR %	Accuracy %
SNVs	176	175	99.43	100	100	99.95	0.57	0	99.96
CNVs	8	8	100	100	100	100	0	0	100
Deletions	4	4	100	100	100	100	0	0	100
Duplications	1	1	100	100	100	100	0	0	100
Fusions	4	4	100	100	100	100	0	0	100

Sensitivity (PPA) = TP/(TP + FN), with BRCA2 p.V1862fs as the only false negative (FN = 1); specificity (NPA) = TN/(TN + FP); precision (PPV) = TP/(TP + FP); NPV = TN/(TN + FN); FNR = FN/(TP + FN); FPR = FP/(FP + TN); accuracy: (TP + TN)/(TP + TN + FP + FN); TP = true positives, TN = true negatives, FP = false positives, FN = false negatives.

**Table 2:** Gene fusions were detected across 107 samples using the CG panel, focusing on key oncogenic driver genes. *ROS1* exhibited the highest number of fusions (22) across 19 samples, followed by *NTRK2* with 12 fusions in 10 samples, highlighting their prevalence. Other genes, such as *ALK*, *MET*, and *NTRK1*, showed fewer fusions (2–4) across 2–4 samples, while *PAX3*, *PAX7*, *RET*, *SS18*, and *YAP1* each appeared in only one sample. This analysis underscores the panel’s ability to detect diverse, clinically relevant fusions.

Gene	Number of Fusions	Fusion Partners	No. of Samples
*ALK*	2	*EML4*, *TNS1*	3
*COL1A1*	3	*RAD51B*, *OCA2*, *PTPRD*	3
*CSF1R*	2	*CAMK2A*, *TMEM132B*	2
*EWSR1*	2	*KCNAB1*, *TMEM212*	2
*FUS*	4	*MAOB, CBS*, *GPR50*, *AUTS2*	4
*MET*	2	*ST7*, *NELL2*	2
*NTRK1*	4	*PLXNA2*, *RPS6KA2*, *DPYSL2*, *POMGNT2*	4
*NTRK2*	12	*RGS6*, *STPG2*, *SHISAL2B*, *GCNT4*, *DPF3*, *DIAPH2*, *ANKS1B*, *PTPRN2*, *S100Z*, *PPARG*, *FRAS1*, *CAMTA1*	10
*NTRK3*	3	*ATRX*, *IPPK*, *DACH1*	3
*PAX3*	4	*AMBP*, *VSNL1*, *CMTR2*, *BPIFB1*	1
*PAX7*	1	*FGFR2*	1
*RET*	1	*NCOA4*	1
*ROS1*	22	*CSMD1*, *EYA1*, *PRKD1*, *KIAA1109*, *KCNIP4*, *CAPZB*, *DSP*, *KCNAB1*, *CDH13*, *MACROD2*, *GPC5*, *DLG2*, *ALDH1A2*, *ASIC2*, *TTC28*, *ADGRL2*, *WDR70*, *FGF12*, *AP2B1*, *STK35*, *NAV2*, *ZBTB20*	19
*SS18*	1	*CBS*	1
*WWTR1*	2	*LTF*, *CBS*	2
*YAP1*	1	*ATXN3*	1

## Data Availability

The data generated and analyzed in this study are included in the published article and its Supplementary Information files. Raw sequencing data (FASTQ), intermediate analysis files, and VCF outputs are not publicly available due to patient privacy restrictions and institutional data protection policies. De-identified data may be made available from the corresponding author upon reasonable request and subject to institutional and ethical approval.

## Supplementary Materials

The following supporting information can be downloaded at https://www.mdpi.com/article/10.3390/biomedicines14071462/s1, Table S1: Distribution of tumor types and sample counts, Table S2: Summary of variants in Acrometrix mutation control reference material, Table S3: Genes covered in the 1Cell.Ai’s gene panel, Table S4: Summary of genes and their corresponding variants analyzed in the study, with copy number variations (CNVs) highlighted in yellow, Table S5: Evaluation of limit of detection of assay for Acrometrix Mutant Hotspot Control at varying VAF and DNA inputs, Table S6: Inter-assay repeatability performance of the assay for various variants, Table S7: Intra-assay accuracy of OncoIndx for samples in triplicates.
