# Supplementary material for: Analytical Validation and Clinical Implementation of a 1080-Gene Comprehensive Genomic Profiling Assay with Integrated Cloud-Based Analysis for Solid Tumor Molecular Oncology
Source: Biomedicines. 2026 Jun 27;14(7):1462. doi: 10.3390/biomedicines14071462 (PMC13403802; doi:10.3390/biomedicines14071462)
Supplement: Supplementary file 1 [file biomedicines-14-01462-s001.zip › biomedicines-4318461-supplementary.pdf]

Table S1. Distribution of Tumor Types and Sample Counts

S.No.	**Tumor type**	**No. of samples**
1	BRAIN	2
2	BREAST	16
3	Cancer of Unknown Primary	1
4	CERVICAL CANCER	1
5	COLON	4
6	Endometrioid carcinoma	4
7	Fallopian Tube Cancer	2
8	Glioblastoma	4
9	Kidney	1
10	Lung metastatic cancer	6
11	liver	1
12	NSCLC	3
13	Melanoma	2
14	Neuroendocrine tumors	2
15	Ovarian	53
16	Pulmonary adenocarcinoma	2
17	Serous carcinoma	2
18	Squamous cell carcinoma	1
	**Total Unique samples**	**107**
	**CAP**	**12**

**Reference material -Acrometrix (Dilutions)+Reference material-Seracare (Dilutions)**	**8**
**Patient samples repeated for Inter and Intra run validation**	**13**
**Total samples analyzed**	**140**

Table S2: Summary of variants in Acrometrix mutation Control reference material

**Acrometrix mutation control**				
**S.No.**	**Gene**	**Mutation CDS**	**Mutation AA**	**21 Variants**
1	*NRAS*	c.182A>G	p.Q61R	
2	*ALK*	c.3522C>A	p.F1174L	
3	*CTNNB1*	c.121A>G	p.T41A	
		c.134C>T	p.S45F	
4	*PIK3CA*	c.1624G>A	p.E542K	
		c.1633G>A	p.E545K	
		c.3140A>G	p.H1047R	
5	*PDGFRA*	c.2525A>T	p.D842V	
6	*KIT*	c.2558G>A	p.W853*(TER)	
7	*FGFR2*	c.755C>G	p.S252W	
8	*KRAS*	c.183A>C	p.Q61H	

		c.35G>A	p.G12D	
9	*AKT1*	c.49G>A	p.E17K	
10	*TP53*	c.818G>A	p.R273H	
		c.743G>A	p.R248Q	
11	*GNAS*	c.601C>T	p.R201C	
12	*EGFR*	c.2235_2249del15	p.E746_A750delELREA	
		c.2573T>G	p.L858R	
		c.2582T>A	p.L861Q	
13	*MET*	c.3757T>G	p.Y1253D	
14	*BRAF*	c.1799T>A	p.V600E	

Table S3. Genes covered in the 1Cell.Ai's gene panel

**S.No.**	**Genelist**	**S.No.**	**Genelist**	**S.No.**	**Genelist**	**S.No.**	**Genelist**	**S.No.**	**Genelist**
**1**	*ABCB1*	**217**	*PASK*	**433**	*CD79A*	**649**	*EZHIP*	**865**	*ZBTB2*
**2**	*ARAF*	**218**	*IKZF3*	**434**	*CD79B*	**650**	*EZR*	**866**	*ZBTB20*
**3**	*ARID2*	**219**	*PDCD1LG2*	**435**	*CD80*	**651**	*FANCE*	**867**	*ZBTB7B*
**4**	*B2M*	**220**	*PIGA*	**436**	*CDC73*	**652**	*FANCF*	**868**	*ZC3H12A*

**5**	*BBC3*	**221**	*PKN1*	**437**	*CDH1*	**653**	*FANCG*	**869**	*ZCCHC12*
**6**	*BIRC3*	**222**	*POLD1*	**438**	*CDH10*	**654**	*FANCI*	**870**	*ZFHX3*
**7**	*CBWD3*	**223**	*PPP4R2*	**439**	*CDH4*	**655**	*FBXO11*	**871**	*ZFP36L1*
**8**	*CIITA*	**224**	*PRKD1*	**440**	*CDK12*	**656**	*FBXO31*	**872**	*ZFP36L2*
**9**	*COL1A1*	**225**	*PTEN*	**441**	*CDK4*	**657**	*FBXW7*	**873**	*ZMYM2*
**10**	*EBF1*	**226**	*PTPRC*	**442**	*CDK6*	**658**	*FCGR2A*	**874**	*ZMYM3*
**11**	*EEF2*	**227**	*RAD51*	**443**	*CDK8*	**659**	*FCGR3A*	**875**	*ZNF133*
**12**	*ECT2L*	**228**	*RASGEF1A*	**444**	*CDKN1A*	**660**	*FGF1*	**876**	*ZNF217*
**13**	*EGFL7*	**229**	*RECQL*	**445**	*CDKN1B*	**661**	*FAS*	**877**	*ZNF24*
**14**	*EML4*	**230**	*RHOA*	**446**	*CDKN1C*	**662**	*ETV5*	**878**	*ZNF384*
**15**	*EPHA5*	**231**	*ROBO1*	**447**	*CDKN2A*	**663**	*FGF7*	**879**	*ZNF703*
**16**	*ERG*	**232**	*RPS6KB2*	**448**	*CDKN2B*	**664**	*FGF8*	**880**	*ZNF750*

**17**	*FANCA*	**233**	*S1PR2*	**449**	*CDKN2C*	**665**	*FLNA*	**881**	*ZNRF3*
**18**	*ETV4*	**234**	*SH2B3*	**450**	*CEBPA*	**666**	*FLT1*	**882**	*ZRANB3*
**19**	*FAM135B*	**235**	*SLC26A3*	**451**	*CENPA*	**667**	*FOXD4L1*	**883**	*ZRSR2*
**20**	*FAT1*	**236**	*TAP2*	**452**	*CFTR*	**668**	*FOXL2*	**884**	*RAF1*
**21**	*FGF23*	**237**	*TERF1*	**453**	*CHD2*	**669**	*FOXO1*	**885**	*TERT*
**22**	*FH*	**238**	*TGIF1*	**454**	*CHD3*	**670**	*FOXO3*	**886**	*TFE3*
**23**	*FGF4*	**239**	*WEE1*	**455**	*CHD4*	**671**	*FOXP1*	**887**	*RAD51B*
**24**	*FGF5*	**240**	*WWTR1*	**456**	*CHD7*	**672**	*FOXQ1*	**888**	*RB1*
**25**	*FGF9*	**241**	*ALK*	**457**	*CHD8*	**673**	*FRK*	**889**	*RECQL4*
**26**	*FGFR1*	**242**	*NTRK2*	**458**	*CHEK1*	**674**	*FGF10*	**890**	*REL*
**27**	*FLT4*	**243**	*CNBD1*	**459**	*CHEK2*	**675**	*FGF12*	**891**	*RHOB*
**28**	*GAB2*	**244**	*ABL2*	**460**	*CIC*	**676**	*FGF14*	**892**	*ROS1*
**29**	*H3C1*	**245**	*ABRAXAS1*	**461**	*BCL2L1*	**677**	*FGF19*	**893**	*RPTOR*
**30**	*H3C8*	**246**	*ACTA2*	**462**	*BCL2L11*	**678**	*FGF2*	**894**	*SALL4*

**31**	*HLA*	**247**	*ACTB*	**463**	*BCL2L12*	**679**	*FLYWCH1*	**895**	*SH2D1A*
**32**	*HRAS*	**248**	*ACVR1*	**464**	*BCL2L2*	**680**	*FOXA1*	**896**	*SLC34A2*
**33**	*IDH1*	**249**	*ACVR1B*	**465**	*BCL6*	**681**	*FOXA2*	**897**	*RINT1*
**34**	*IFNGR1*	**250**	*ACVR2A*	**466**	*BCL7A*	**682**	*GAB1*	**898**	*RIT1*
**35**	*IL2RG*	**251**	*ADGRA2*	**467**	*BCL9*	**683**	*GATA1*	**899**	*TAPBP*
**36**	*IL4R*	**252**	*ADGRB1*	**468**	*BCLAF1*	**684**	*GATA2*	**900**	*THADA*
**37**	*INPPL1*	**253**	*AGO1*	**469**	*BCOR*	**685**	*GATA3*	**901**	*WIF1*
**38**	*JAK2*	**254**	*AGO2*	**470**	*BCORL1*	**686**	*GATA4*	**902**	*RAD51C*
**39**	*ITPKB*	**255**	*AJUBA*	**471**	*BCR*	**687**	*GATA6*	**903**	*RAD51D*
**40**	*IGF1R*	**256**	*AKT1*	**472**	*CPS1*	**688**	*GEM*	**904**	*RAD52*
**41**	*IL6ST*	**257**	*AKT2*	**473**	*CRBN*	**689**	*GEN1*	**905**	*RAD54B*
**42**	*IRF2*	**258**	*AKT3*	**474**	*CREB3L3*	**690**	*GID4*	**906**	*RAD54L*
**43**	*IRF4*	**259**	*ALB*	**475**	*CREBBP*	**691**	*GLI1*	**907**	*RANBP17*

**44**	*JAK3*	**260**	*ALOX12B*	**476**	*CRKL*	**692**	*GLI2*	**908**	*RHPN2*
**45**	*IDH2*	**261**	*AMER1*	**477**	*CRLF1*	**693**	*GLIS2*	**909**	*RPA1*
**46**	*IKBKE*	**262**	*ANKRD11*	**478**	*CRLF2*	**694**	*GNA11*	**910**	*RRAGC*
**47**	*ING1*	**263**	*ANKRD26*	**479**	*CRTC1*	**695**	*GNA12*	**911**	*SAMD9*
**48**	*IDO1*	**264**	*APC*	**480**	*CSDE1*	**696**	*GNA13*	**912**	*SHH*
**49**	*IL2*	**265**	*APH1A*	**481**	*CSF1R*	**697**	*GNAI2*	**913**	*SLFN11*
**50**	*IFNAR1*	**266**	*APLNR*	**482**	*CSF3R*	**698**	*GNAQ*	**914**	*TBL1XR1*
**51**	*IL2RB*	**267**	*APOB*	**483**	*CSNK1A1*	**699**	*GNAS*	**915**	*TET1*
**52**	*INHA*	**268**	*AR*	**484**	*CTC1*	**700**	*GNB1*	**916**	*THRAP3*
**53**	*IRF6*	**269**	*ARHGAP26*	**485**	*CTCF*	**701**	*GPC3*	**917**	*RICTOR*
**54**	*IRF8*	**270**	*ARHGAP35*	**486**	*CTDNEP1*	**702**	*GPS2*	**918**	*RPL10*
**55**	*IRS1*	**271**	*ARHGEF10*	**487**	*CTLA4*	**703**	*GREM1*	**919**	*RRAS*
**56**	*IRS2*	**272**	*ARHGEF12*	**488**	*CTNNA1*	**704**	*GRIN2A*	**920**	*SAMD9L*

**57**	*IRS4*	**273**	*ARID1A*	**489**	*CTNNB1*	**705**	*GRIN2D*	**921**	*SHOC2*
**58**	*ITGAM*	**274**	*ARID1B*	**490**	*CTNND1*	**706**	*GRM3*	**922**	*SLIT2*
**59**	*JARID2*	**275**	*ARID4B*	**491**	*CTR9*	**707**	*GSK3B*	**923**	*TBX3*
**60**	*KDM4C*	**276**	*ARID5B*	**492**	*CUL1*	**708**	*GTF2I*	**924**	*TET2*
**61**	*KEAP1*	**277**	*ASMTL*	**493**	*CUL3*	**709**	*GTSE1*	**925**	*TIPARP*
**62**	*KIF1A*	**278**	*ASXL1*	**494**	*CUL4A*	**710**	*H1-2*	**926**	*SLX4*
**63**	*KIT*	**279**	*ASXL2*	**495**	*CUL4B*	**711**	*H1-3*	**927**	*SMAD2*
**64**	*KLF2*	**280**	*ATF7IP*	**496**	*CUX1*	**712**	*H1-4*	**928**	*SMAD3*
**65**	*KRAS*	**281**	*ATM*	**497**	*CXCR4*	**713**	*H19*	**929**	*SMAD4*
**66**	*LRRK2*	**282**	*ATP6AP1*	**498**	*CYLD*	**714**	*H2AC11*	**930**	*SMARCA1*
**67**	*LDB1*	**283**	*ATP6V1B2*	**499**	*CYP17A1*	**715**	*H2AC16*	**931**	*SMARCA2*
**68**	LEF1	**284**	*ATR*	**500**	*CYP19A1*	**716**	*H2AC17*	**932**	*SMARCA4*
**69**	LEMD2	**285**	*ATRX*	**501**	*CYP2D6*	**717**	*H2AC6*	**933**	*SMARCAL1*

70	LMO1	286	ATXN3	502	CYSLTR2	718	H2BC11	934	SMARCB1
71	LRP5	287	ATXN7	503	DACH1	719	H2BC12	935	SMARCD1
72	LRP6	288	AURKA	504	DAXX	720	H2BC17	936	SMARCE1
73	LTK	289	AURKB	505	DAZAP1	721	H2BC4	937	SMC1A
74	LUC7L2	290	AXIN1	506	DCSTAMP	722	H2BC5	938	SMC3
75	MAGED1	291	AXIN2	507	DCUN1D1	723	H3-3A	939	SMO
76	MAP3K6	292	AXL	508	DDB2	724	H3-3B	940	SMYD3
77	MDM4	293	BAP1	509	DDR1	725	H3-4	941	SNCAIP
78	MIB1	294	BCL11B	510	DDR2	726	H3-5	942	SOCS1
79	MR1	295	BMPR1A	511	DDX3X	727	H3C13	943	SOCS2
80	MTAP	296	CCL2	512	DDX41	728	H3C14	944	SOCS3
81	MYH11	297	CKS1B	513	DEK	729	H3C15	945	SOS

**82**	NEGR1	**298**	*COL7A1*	**514**	*DHX9*	**730**	*H3C2*	**946**	*SOX10*
**83**	NOD1	**299**	*CNOT9*	**515**	*DIAPH2*	**731**	*H3C3*	**947**	*SOX17*
**84**	NSD3	**300**	*EEF1A1*	**516**	*DICER1*	**732**	*H3C4*	**948**	*SOX2*
**85**	NUP93	**301**	*EGLN1*	**517**	*DIS3*	**733**	*H3C6*	**949**	*SOX9*
**86**	PARP3	**302**	*ENG*	**518**	*DIS3L2*	**734**	*H3C7*	**950**	*SP140*
**87**	PCBP1	**303**	*EPHB1*	**519**	*DKC1*	**735**	*HDAC4*	**951**	*SPEN*
**88**	PCLO	**304**	*ESR1*	**520**	*DKK*	**736**	*HDAC7*	**952**	*SPOP*
**89**	PHF6	**305**	*FANCC*	**521**	*DMD*	**737**	*HDAC9*	**953**	*SPRED1*
**90**	PIK3R2	**306**	*FANCL*	**522**	*DNAJB1*	**738**	*HGF*	**954**	*SPRTN*
**91**	*PMS1*	**307**	*FGR*	**523**	*DNM2*	**739**	*HIF1A*	**955**	*SPTA1*
**92**	*PPM1D*	**308**	*FLCN*	**524**	*DNMT1*	**740**	*HLA-A*	**956**	*SPTAN1*
**93**	*PRKCB*	**309**	*FRS2*	**525**	*DNMT3A*	**741**	*HLA-B*	**957**	*SRC*

**94**	*PSMB5*	**310**	*FGFR3*	**526**	*DNMT3B*	**742**	*HLA-C*	**958**	*SRP72*
**95**	*PTPN14*	**311**	*FUS*	**527**	*DOCK8*	**743**	*HLA-F*	**959**	*SRSF2*
**96**	*RAB35*	**312**	*GADD45B*	**528**	*DOT1L*	**744**	*HLA-G*	**960**	*SS18*
**97**	*RAC2*	**313**	*H3C11*	**529**	*DROSHA*	**745**	*HLTF*	**961**	*SSBP2*
**98**	*RANBP2*	**314**	*HDAC1*	**530**	*DTX1*	**746**	*HMGA2*	**962**	*STAG1*
**99**	*RBM10*	**315**	*HSP90AA1*	**531**	*DUSP2*	**747**	*HNF1A*	**963**	*STAG2*
**100**	*RELN*	**316**	*IGF1*	**532**	*DUSP22*	**748**	*HNRNPK*	**964**	*STAT1*
**101**	*RELA*	**317**	*IRF1*	**533**	*DUSP4*	**749**	*HOXA11*	**965**	*STAT2*
**102**	*RFC1*	**318**	*IKZF2*	**534**	*DUSP9*	**750**	*HOXB13*	**966**	*STAT3*
**103**	*RNF111*	**319**	*INO80*	**535**	*E2F3*	**751**	*HUWE1*	**967**	*STAT4*
**104**	*RPS6KA3*	**320**	*JUN*	**536**	*IL10*	**752**	*ICOS*	**968**	*STAT5A*

**105**	*RUNX1T1*	**321**	*KDM5C*	**537**	*INPP4B*	**753**	*ICOSLG*	**969**	*STAT5B*
**106**	*SF3A1*	**322**	*KIF5B*	**538**	*INPP5D*	**754**	*ID3*	**970**	*STAT6*
**107**	*SHQ1*	**323**	*KLF4*	**539**	*KAT6A*	**755**	*MLH3*	**971**	*STK11*
**108**	*TAF1*	**324**	*LAG3*	**540**	*KAT6B*	**756**	*MSI2*	**972**	*STK19*
**109**	*TAF15*	**325**	*LZTR1*	**541**	*KBTBD4*	**757**	*MYBL1*	**973**	*STK40*
**110**	*TENT5C*	**326**	*MALT1*	**542**	*KDM2B*	**758**	*NCOA3*	**974**	*SUFU*
**111**	*TFG*	**327**	*MAPK1*	**543**	*KDM6B*	**759**	*NFKBIA*	**975**	*SUSD2*
**112**	*VTCN1*	**328**	*MECOM*	**544**	*KDR*	**760**	*NR4A3*	**976**	*SUZ12*
**113**	*WNK2*	**329**	*MIR142*	**545**	*KLHL6*	**761**	*NUDT15*	**977**	*SYK*
**114**	*NOTCH2*	**330**	*MRTFA*	**546**	*KLLN*	**762**	*PAK3*	**978**	*TCL1A*
**115**	*ABL1*	**331**	*MUC17*	**547**	*KMT2A*	**763**	*PAX8*	**979**	*RPL22*
**116**	*ARFRP1*	**332**	*MYO18A*	**548**	*KMT2B*	**764**	*PDK1*	**980**	*RRAS2*
**117**	*ARID3A*	**333**	*NF1*	**549**	*KMT2C*	**765**	*PIK3CA*	**981**	*SAMHD1*

**118**	*BABAM1*	**334**	*NT5E*	**550**	*KMT2D*	**766**	*PLCG2*	**982**	*RPL5*
**119**	*BCL10*	**335**	*NUTM1*	**551**	*KNSTRN*	**767**	*POLR2A*	**983**	*RSPO2*
**120**	*BLM*	**336**	*PARPBP*	**552**	*LATS2*	**768**	*PREX2*	**984**	*SBDS*
**121**	*CCDC6*	**337**	*PDCD11*	**553**	*LCK*	**769**	*PRPF8*	**985**	*TCF12*
**122**	*CILK1*	**338**	*PICALM*	**554**	*MAD2L2*	**770**	*PTP4A1*	**986**	*TET3*
**123**	*COL5A1*	**339**	*PIM1*	**555**	*MAF*	**771**	*PTPRT*	**987**	*TLL2*
**124**	*ELF3*	**340**	*PNRC1*	**556**	*MAFB*	**772**	*MLH1*	**988**	*TCF3*
**125**	*EED*	**341**	*PPP2R2A*	**557**	*MAMLD1*	**773**	*MSH6*	**989**	*TLR4*
**126**	*EGFR*	**342**	*PRKCI*	**558**	*MAP2K1*	**774**	*MYB*	**990**	*RPS15*
**127**	*EMSY*	**343**	*PTCH2*	**559**	*MAP2K2*	**775**	*NCOA2*	**991**	*RSPO3*
**128**	*EPHA7*	**344**	*PTPN6*	**560**	*MAP2K4*	**776**	*NFE2L2*	**992**	*SCAF4*
**129**	*ERRFI1*	**345**	*RAC1*	**561**	*MAP3K1*	**777**	*NPRL2*	**993**	*RPS20*

**130**	*FANCB*	**346**	*RAD50*	**562**	*MAP3K13*	**778**	*NTRK3*	**994**	*RTEL1*
**131**	*FAF1*	**347**	*RASA1*	**563**	*MAP3K14*	**779**	*PAK1*	**995**	*SCG5*
**132**	*FASLG*	**348**	*RBM38*	**564**	*MAP3K4*	**780**	*PAX7*	**996**	*TCF7L2*
**133**	*FGF3*	**349**	*RET*	**565**	*MAST1*	**781**	*PDGFRB*	**997**	*TFEB*
**134**	*FHIT*	**350**	*RHEB*	**566**	*MAST2*	**782**	*PIK3C3*	**998**	*TLR9*
**135**	*FGF6*	**351**	*RNF43*	**567**	*MAX*	**783**	*PLCG1*	**999**	*RPS3A*
**136**	*FLT3*	**352**	*RPS6KB1*	**568**	*MBD4*	**784**	*POLQ*	**1000**	*RUNX1*
**137**	*FGFR2*	**353**	*RYBP*	**569**	*MC1R*	**785**	*PRDM14*	**1001**	*SDC4*
**138**	*FUBP1*	**354**	*SGK1*	**570**	*MCL1*	**786**	*PRPF40B*	**1002**	*TLX3*
**139**	*GABRA6*	**355**	*SIRPA*	**571**	*MDC*	**787**	*PTMA*	**1003**	*TEK*
**140**	*H3C10*	**356**	*TAP1*	**572**	*MDM2*	**788**	*PTPRS*	**1004**	*TDG*
**141**	*HAVCR2*	**357**	*TERC*	**573**	*MEF2B*	**789**	*MPL*	**1005**	*TCL1B*

**142**	*HSD3 B1*	**358**	*TGFBR 2*	**574**	*MEF2C*	**790**	*MT1JP*	**100 6**	*TMEM127*
**143**	*IFNGR 2*	**359**	*WDR90*	**575**	*MEF2D*	**791**	*MYD8 8*	**100 7**	*SDHA*
**144**	*IL3*	**360**	*WT1*	**576**	*MEN1*	**792**	*NECTI N4*	**100 8**	*SDHAF2*
**145**	*INSR*	**361**	*NTRK1*	**577**	*MERT K*	**793**	*NKX3- 1*	**100 9**	*SDHB*
**146**	*JAK1*	**362**	*BARD1*	**578**	*MET*	**794**	*NSD2*	**101 0**	*SDHC*
**147**	*IGF2*	**363**	*BCL2*	**579**	*MGA*	**795**	*NUP21 4*	**101 1**	*SDHD*
**148**	*IL7R*	**364**	*BRAF*	**580**	*MGMT*	**796**	*PARP2*	**101 2**	*SERP2*
**149**	*IKZF1*	**365**	*CCN6*	**581**	*MKNK 1*	**797**	*PGR*	**101 3**	*SERPINA1*
**150**	*INHBA*	**366**	*CLIP1*	**582**	*MSH3*	**798**	*PIK3R 1*	**101 4**	*SERPINB3*
**151**	*ITK*	**367**	*COP*	**583**	*MUTY H*	**799**	*PML*	**101 5**	*SERPINB4*
**152**	*JAZF1*	**368**	*EGR1*	**584**	*NBN*	**800**	*PPAR G*	**101 6**	*SESN2*

**153**	*KDM5A*	**369**	*EP300*	**585**	*NFE2*	**801**	*PRKCA*	**1017**	*SESN3*
**154**	*KEL*	**370**	*EPHB4*	**586**	*NPM1*	**802**	*PSIP1*	**1018**	*SETBP1*
**155**	*KIF1B*	**371**	*ETV6*	**587**	*PAG1*	**803**	*PTPN13*	**1019**	*SETD1B*
**156**	*KLF3*	**372**	*FANCD2*	**588**	*PAX5*	**804**	*MLLT3*	**1020**	*SETD2*
**157**	*KRT222*	**373**	*FANCM*	**589**	*PDGFRA*	**805**	*MST1R*	**1021**	*SETDB1*
**158**	*LTB*	**374**	*FLI1*	**590**	*PIK3C2G*	**806**	*MYCN*	**1022**	*SETDB2*
**159**	*LIFR*	**375**	*FGFR4*	**591**	*PLCB4*	**807**	*NCSTN*	**1023**	*SF1*
**160**	*LRP1B*	**376**	*FYN*	**592**	*POLH*	**808**	*NKX2-1*	**1024**	*TMEM30A*
**161**	*LYN*	**377**	*GALNT12*	**593**	*PRDM1*	**809**	*NSD1*	**1025**	*TMPRSS2*
**162**	*MAGI2*	**378**	*H3C12*	**594**	*PRKN*	**810**	*NUP133*	**1026**	*TMSB4X*
**163**	*MAP3K7*	**379**	*HDAC2*	**595**	*PTK2B*	**811**	*PARP1*	**1027**	*TMSB4XP8*

**164**	*MEAF6*	**380**	*HLA-E*	**596**	*PTPRO*	**812**	*PC*	**1028**	*TNFAIP3*
**165**	*MIDEAS*	**381**	*HSP90AB1*	**597**	*MKI67*	**813**	*PGBD5*	**1029**	*TNFRSF11ATNFRSF14*
**166**	*MRE11*	**382**	*CMTR2*	**598**	*MSH2*	**814**	*PIK3CG*	**1030**	*TNFRSF17*
**167**	*MTOR*	**383**	*BRCA1*	**599**	*MUSK*	**815**	*PMAIP1*	**1031**	*TNFRSF18*
**168**	*MYH9*	**384**	*BRCA2*	**600**	*NADK*	**816**	*POU2F2*	**1032**	*TNFRSF4*
**169**	*NEIL2*	**385**	*BRCC3*	**601**	*NFATC2*	**817**	*PRKAR1A*	**1033**	*TNFRSF9*
**170**	*NOTCH1*	**386**	*BRD3*	**602**	*NOTCH4*	**818**	*PRSS8*	**1034**	*TOP*
**171**	*NT5C2*	**387**	*BRD4*	**603**	*PABPC1*	**819**	*PTPN11*	**1035**	*TOP2A*
**172**	*NUP98*	**388**	*BRD7*	**604**	*PAX3*	**820**	*MLLT10*	**1036**	*TP53*
**173**	*PARP4*	**389**	*BRINP3*	**605**	*PDGFB*	**821**	*MST1*	**1037**	*TP53BP1*
**174**	*PDCD1*	**390**	*BRIP1*	**606**	*PIK3C2B*	**822**	*MYCL*	**1038**	*TP63*

**175**	*PHOX2B*	**391**	*BRSK1*	**607**	*PLAG1*	**823**	*NCOR2*	**1039**	*TPMT*
**176**	*PIK3R3*	**392**	*BTG1*	**608**	*POLE*	**824**	*NIPBL*	**1040**	*TPTE2*
**177**	*PMS2*	**393**	*BTG2*	**609**	*PPP6C*	**825**	*NUMBL*	**1041**	*TRAF2*
**178**	*PPP2R1A*	**394**	*BTK*	**610**	*PRKDC*	**826**	*PALB2*	**1042**	*TRAF3*
**179**	*PRKCD*	**395**	*BTLA*	**611**	*PTK2*	**827**	*PBRM1*	**1043**	*TRAF5*
**180**	*PTCH1*	**396**	*BUB1B*	**612**	*PTPRD*	**828**	*PDS5B*	**1044**	*TRAF7*
**181**	*PTPN2*	**397**	*C3orf70*	**613**	*EGR2*	**829**	*PIK3CD*	**1045**	*TRIP13*
**182**	*RABEP1*	**398**	*C8orf34*	**614**	*EGR3*	**830**	*PLXNB2*	**1046**	*TRPA1*
**183**	*RAD21*	**399**	*CACNA1A*	**615**	*EIF1AX*	**831**	*POT1*	**1047**	*TSC1*
**184**	*RARA*	**400**	*CACNA1D*	**616**	*EIF3E*	**832**	*PRKACA*	**1048**	*TSC2*
**185**	*RBM15*	**401**	*CAD*	**617**	*EIF4A2*	**833**	*PRSS1*	**1049**	*TSHR*

**186**	*REST*	**402**	*CALR*	**618**	*EIF4E*	**834**	*PTPN1*	**1050**	*TSLP*
**187**	*RGPD3*	**403**	*CAMTA1*	**619**	*ELANE*	**835**	*MLLT1*	**1051**	*TUSC3*
**188**	*RNF139*	**404**	*CARD11*	**620**	*ELOC*	**836**	*MSMB*	**1052**	*TXNIP*
**189**	*RPS6KA4*	**405**	*CARM1*	**621**	*ELP2*	**837**	*MYC*	**1053**	*TYK2*
**190**	*RXRA*	**406**	*CASP8*	**622**	*EPAS1*	**838**	*NCOR1*	**1054**	*TYRO3*
**191**	*SF3B1*	**407**	*CBFA2T3*	**623**	*EPC1*	**839**	*NFKBIE*	**1055**	*U2AF1*
**192**	*SIN3A*	**408**	*CBFB*	**624**	*EPCAM*	**840**	*NRAS*	**1056**	*U2AF2*
**193**	*TAL1*	**409**	*CBL*	**625**	*EPHA2*	**841**	*NUF2*	**1057**	*UBE2T*
**194**	*TENT5D*	**410**	*CBLB*	**626**	*EPHA3*	**842**	*PAK5*	**1058**	*UBR5*
**195**	*TGFBR1*	**411**	*CBLC*	**627**	*EGP1*	**843**	*PAXIP1*	**1059**	*UNCX*
**196**	*WAS*	**412**	*CCNB3*	**628**	*ERBB2*	**844**	*PDPK1*	**1060**	*USP6*

**197**	*WRN*	**413**	*CCND1*	**629**	*ERBB3*	**845**	*PIK3CB*	**1061**	*USP8*
**198**	*NRG1*	**414**	*CCND2*	**630**	*ERBB4*	**846**	*PLK2*	**1062**	*USP9X*
**199**	*INPP4A*	**415**	*CCND3*	**631**	*ERCC1*	**847**	*POLRMT*	**1063**	*VAV1*
**200**	*KANSL1*	**416**	*CCNE1*	**632**	*ERCC2*	**848**	*PRF1*	**1064**	*VEGFA*
**201**	*KDM6A*	**417**	*CCR2*	**633**	*ERCC3*	**849**	*PRPS1*	**1065**	*VHL*
**202**	*KIR3DL1*	**418**	*CCR4*	**634**	*ERCC4*	**850**	*PTPDC1*	**1066**	*DMA*
**203**	*KLF5*	**419**	*CCR5*	**635**	*ERCC5*	**851**	*QKI*	**1067**	*DRB1*
**204**	*LATS1*	**420**	*CCT6B*	**636**	*ERCC6*	**852**	*XBP1*	**1068**	*DMB*
**205**	*MACF1*	**421**	*CD19*	**637**	*ERF*	**853**	*XIAP*	**1069**	*DRB5*
**206**	MAML2	**422**	CD22	**638**	EWSR1	**854**	XPA	**1070**	*DOA*
**207**	MAPK3	**423**	CD27	**639**	EXO1	**855**	XPC	**1071**	*DRB6*

**208**	MED1 2	**424**	CD274	**640**	EXOS C6	**856**	XPO1	**107 2**	*DOB*
**209**	MITF	**425**	CD276	**641**	EXT1	**857**	XRCC 1	**107 3**	*DPA1*
**210**	MRTF B	**426**	CD28	**642**	EXT2	**858**	XRCC 2	**107 4**	*DPB1*
**211**	MUC6	**427**	CD33	**643**	ESRR A	**859**	XRCC 3	**107 5**	*DPB2*
**212**	MYOD 1	**428**	CD36	**644**	ETNK1	**860**	YAP1	**107 6**	*DQA1*
**213**	NF2	**429**	CD40	**645**	ETS1	**861**	YEATS 4	**107 7**	*DQA2*
**214**	NOTC H3	**430**	CD58	**646**	ETV1	**862**	YES1	**107 8**	*DQB1*
**215**	NTHL1	**431**	CD70	**647**	EZH1	**863**	YWHA E	**107 9**	*DQB2*
**216**	P2RY8	**432**	CD74	**648**	EZH2	**864**	YY1AP 1	**108 0**	*DRA*

Table S4: Summary of genes and their corresponding variants analyzed in the study, with copy number variations (CNVs) highlighted in yellow.

S.No.	Gene	Variant
1	*ALK*	p.R1275Q
2	*AR*	p.V132I
3	*ARAF*	p.D286N
4	*ARID2*	p.I416M
5	*ARID5B*	p.R948G
6	*ATM*	p.T2438I
		p.A112T
		p.C107Y
		p.1570A
		p.D1853N
		M1040V
		p.F858L
		P1054R
		p.R717Q
7	*ATR*	p.Y656H
8	*AXL*	p.Q124E
9	*BARD1*	V507M
		R378S
		C557S

		P24S
10	*BRAF*	p.V600E
		p.V600K
11	*BRCA1*	p.Q491*
		p.E23Vfs*17
		p.L156I
		p.M1673I
		p.K65T
		p.D693N
		p.K1183R
		p.P871L
		p.S1613G
		p.V191I
12	*BRCA2*	p.Q147R
		p.N372H
		p.N991D
		p.I3412V
		p.E1232K
		p.D1420Y
		p.V1862fs
13	*CDK12*	p.Q1291fs
		p.T1447S
		p.I1131V

14	*CDKN2A*	p.A148T
15	*CHEK1*	p.K166N
		p.I471V
16	*CHEK2*	p.I157T
17	*CSF3R*	p.W542R
18	*CTNNB1*	p.H737R
		p.S37C
		p.S33C
		p.T41A
		p.D32N
		p.M98V
19	*DAXX*	p.N614S
20	*EGFR*	p. E746_A750del
		p. T790M
		p.G719X
		p.L861Q
		p.S768I
		p.S464L
		p.R521K
		p.L858R
21	*ERCC4*	p.R726H
22	*ESR1*	p.K303R
23	*ERBB2*	p. Y772_A775dup

		p.I655V
		p.W452C
		p.D30E
24	*FANCA*	p.A906T
		p.A181V
		p.N8K
		p.G809D
		p.T1328A
		p.T266A
		p.V6D
		p.S1088F
25	*FBXW7*	p.R465H
26	*FGFR2*	p.P804S
27	*FGFR3*	p.S249C
		p.E322G
		p.R399H
		p.R568Q
		p.P449S
28	*FLT4*	p.K117E
29	*GATA4*	p.R195Q
		p.P14R
30	*IDH1*	p.R132H
		p.V178I

		p.V71I
		p.R132G
		p.R132L
31	*IDH2*	p.R140W
		p.R172M
32	*KDM5A*	p.R1239W
33	*KDR*	p.E628D
34	*KIT*	p.A502-Y503insAY
		p.P468Q
		p.D816V
		p.L793F
		p.V13A
		p.C691Y
		p.M541L
		p.M722V
35	*KMT2C*	p.A3825P
36	*KRAS*	p.G13D
		p.A146P
		p.G12W
		p.G13E
		p.Q61H
		p.G12D
		p.G12V

		p. G12C
37	*MET*	p.D1028Y
		p.E168D
38	*MSH6*	p.G39E
39	*MLH1*	p.I47T
		p.I219V
40	*MYOD1*	p.L122R
41	*NOTCH1*	p.G2245V
42	*NCOR1*	p.G2320V
43	*NRAS*	p.Q61R
		p.G12A
		p.Q61L
44	*PALB2*	p.S380Rfs*43
		p.E672Q
45	*PDGFRA*	p.I1025M
		p.A820S
		p.L593P
		p. P1021T
		p.S566_E571 delinsR
		p.G79D
		p.E938K
		p.S478P
		p.E263*

46	*PIK3CA*	p.E545K
		p.E542K
		p.H1047R
		p.Q546E
47	*PMS2*	p. D286H
		p.V380G
48	*POLE*	p.R1508H
		p.P286R
49	*RAD15*	p.T27I
50	*RARA*	p.P76S
51	*RAD51B*	p.R159C
		p.L172W
52	*RAD51D*	p.R165Q
53	*RAD51C*	p.T287A
54	*RB1*	p. P20L
55	*RET*	p.G691S
		p.R982C
56	*RFWD2*	p.S105I
57	*RAD54L*	p.S636T)
58	*SMARC4*	p.K467N
59	*TP53*	p. H178Sfs*69
		p.V173L
		p.R175H

		p.Q136P
		p.M237I
		p.Q317*
		p.S241Y,
		p.P190L
		p.R337L
		p.E339
		p.R280S
		p.R213*
		p.L194F
		p.C242F
		p.R249T
		p.F212Sfs*3
		p.A161D
		p.R248W
		p.Y234C
		p.G245C
		p.H179R
60	*VHL*	p.L188Q
61	*MYCN*	
62	*CDK4*	
63	*MDM2*	
64	*MYC*	

65	*COL1A1*	**Gene involved in fusions**
66	*CSF1 (R)*	
67	*EWSR1*	
68	*FUS*	
69	*NTRK1*	
70	*NTRK2*	
71	*NTRK3*	
72	*PAX3*	
73	*PAX7*	
74	*ROS1*	
75	*SS18*	
76	*WWTR1*	
77	*YAP-1*	
78	*NCOA4*	
79	*ST7*	
80	*TNS1*	
81	*EML4*	

Table S5. Evaluation of Limit of detection of assay for Acrometrix Mutant Hotspot Control at Varying VAF and DNA Inputs

Acrometrix Mutant Hotspot control								
**Gene**	**Mutation CDS**	**Mutation AA**	**Target Frequency**	**100% (100 ng)**	**50% (50 ng)**	**25% (25 ng)**	**10% (10 ng)**	**5% (5ng)**
NRAS	c.182A>G	p.Q61R	5-15%	✓	✓	✓	✓	✓
ALK	c.3522C>A	p.F1174L		✓	✓	✓	✓	-
CTNNB1	c.121A>G	p.T41A		✓	✓	✓	✓	✓
	c.134C>T	p.S45F		✓	✓	✓	✓	✓
PIK3CA	c.1624G>A	p.E542K		✓	✓	-	-	-
	c.1633G>A	p.E545K		✓	✓	-	-	-
	c.3140A>G	p.H1047R		✓	✓	✓	✓	-
PDGFRA	c.2525A>T	p.D842V		✓	✓	✓	✓	✓
KIT	c.2558G>A	p.W853*(TER)		✓	✓	✓	✓	✓
FGFR2	c.755C>G	p.S252W		✓	✓	✓	✓	✓
KRAS	c.183A>C	p.Q61H		✓	✓	✓	✓	✓
	c.35G>A	p.G12D		✓	✓	✓	✓	✓
AKT1	c.49G>A	p.E17K		✓	✓	✓	✓	✓
TP53	c.818G>A	p.R273H		✓	✓	✓	✓	✓
	c.743G>A	p.R248Q		✓	✓	✓	✓	✓
GNAS	c.601C>T	p.R201C		✓	✓	✓	✓	✓

EGFR	c.2235_2249del15	p.E746_A750delELREA	15-35%	✓	✓	✓	✓	-
	c.2573T>G	p.L858R		✓	✓	✓	✓	✓
	c.2582T>A	p.L861Q		✓	✓	✓	✓	✓
MET	c.3757T>G	p.Y1253D		✓	✓	✓	✓	✓
BRAF	c.1799T>A	p.V600E		✓	✓	✓	✓	✓
**14 genes**		**21 variants**		**21**	**21**	**19**	**18**	**16**

Table S6. Inter-assay repeatability performance of the assay for various variants.

	Sample	Run ID	Sample ID	Expected Variants	Observed Variants
**Inter Run analysis**	8F-SP22-8769-B1	Run-1	4_8F-SP22-8769-B1-Rep-3	*KRAS* G12D	*KRAS* G12D
		Run-3	21_8F_SP22-8769-B1-Rep4_InterRun		
		Run-4	47_8F_SP22-8769-B1-Rep4_InterRun		
	HRD1_SP20-12588-B5	Run-2	37_HRD1_SP-20-12588-B5-REP-3	*BRCA1* p.E23Vfs*17, *KIT* p.P468Q, *FLT4* p.K117E. *DAXX* p.N614S, *KDR* p.E628D, *PDGFRA* p.E263*. p1021T, *PMS2* p.V380G, *POLE* p.R1508H, *RB1* p. P20L,	*BRCA1* p.E23Vfs*17, *KIT* p.P468Q, *FLT4* p.K117E. *DAXX* p.N614S, *KDR* p.E628D, *PDGFRA* p.E263*. p1021T, *PMS2* p.V380G, *POLE* p.R1508H, *RB1* p. P20L, *RFWD2* p.S105I, *TP53* p.Y234C
		Run-3	46_HRD1_SP20-12588-B5-REP-4_IR2		
		Run-4	61_HRD1_SP20-12588-B5-REP-5_InterRun		

				RFWD2 p.S105I, *TP53* p.Y234C	
	TA5_SP22-7596-B1	RUN-1	16_TA5_SP22-7596-B1-REP-1	*KRAS* p.Q61H, *CTNNB1* p.D32N	*KRAS* p.Q61H, *CTNNB1* p.D32N
		RUN-4	63_TA5_SP22-7596-B1-REP-2		
	TA6_SP22-15835-A7	RUN-1	17_TA6_SP22-15835-A7	*CTNNB1* p.T41A	*CTNNB1* p.T41A
		RUN-4	64_TA6_SP22-15835-A7		
	TA12_SP20-475-A7	RUN-1	18_TA12_SP20-475-A7	*NRAS* p.Q61R	*NRAS* p.Q61R
		RUN-4	66_TA12_SP20-475-A7		
	TA16_SP22-6870-B1	RUN-1	19_TA16_SP22-6870-B1	*PDGFRA* p.A820S, p.L593P	*PDGFRA* p.A820S, p.L593P
		RUN-4	67_TA16_SP22-6870-B1		
	20_TA18_SP22-6326-A1	RUN-1	20_TA18_SP22-6326-A1	*FGFR3* p.S249C	*FGFR3* p.S249C
		RUN-4	68_TA18_SP22-6326-A1		

Table S7. Intra-assay accuracy of OncoIndx for samples in triplicates.

	**Sample**	**Run ID**	**Sample ID**	**Expected Variants**	**Observed Variants**
**Intra Run Analysis**	8F-SP22-8769-B1	Run-1	2_8F-SP22-8769-B1-Rep-1	*KRAS* G12D	*KRAS* G12D
			3_8F-SP22-8769-B1-Rep-2		

			4_8F-SP22-8769-B1-Rep-3		
	HRD1_SP-20-12588-B5-	Run-2	35_HRD1_SP-20-12588-B5-REP-1	*BRCA1* p.E23Vfs*17, *KIT* p.P468Q, *FLT4* p.K117E. *DAXX* p.N614S, *KDR* p.E628D, *PDGFRA* p.E263*. p1021T, *PMS2* p.V380G, *POLE* p.R1508H, *RB1* p. P20L, *RFWD2* p.S105I, *TP53* p.Y234C	*BRCA1* p.E23Vfs*17, *KIT* p.P468Q, *FLT4* p.K117E. *DAXX* p.N614S, *KDR* p.E628D, *PDGFRA* p.E263*. p1021T, *PMS2* p.V380G, *POLE* p.R1508H, *RB1* p. P20L, *RFWD2* p.S105I, *TP53* p.Y234C
